# Tænia in an Infant Five Days Old

**Published:** 1872-01

**Authors:** 


Tænia in an Infant Five Days Old.—In the Detroit Review of Medicine and Pharmacy, January, 1872, Dr. S. G. Armour reports quite a unique case occurring in the Long Island College Hospital. An infant three days old was attacked with *trismus;* two days after the attack, and five days after birth, the infant commenced passing well-matured *tænia solium*, and continued to pass segment after segment for five or six days, when the trismus ceased, and the child had a good recovery. Two months after the birth of the child, the mother was put under treatment for tænia, and passed seventy or eighty segments. How did the parasitic embryo gain entrance to the intestinal canal of the fœtus in utero?

